# A Two-Stage Attention-Based Hierarchical Transformer for Turbofan Engine Remaining Useful Life Prediction

**DOI:** 10.3390/s24030824

**Published:** 2024-01-26

**Authors:** Zhengyang Fan, Wanru Li, Kuo-Chu Chang

**Affiliations:** Department of Systems Engineering and Operations Research, George Mason University, Fairfax, VA 22030, USA; wli15@gmu.edu

**Keywords:** two-stage attention, multiscale transformer, remaining useful life prediction, turbofan aircraft engine

## Abstract

The accurate estimation of the remaining useful life (RUL) for aircraft engines is essential for ensuring safety and uninterrupted operations in the aviation industry. Numerous investigations have leveraged the success of the attention-based Transformer architecture in sequence modeling tasks, particularly in its application to RUL prediction. These studies primarily focus on utilizing onboard sensor readings as input predictors. While various Transformer-based approaches have demonstrated improvement in RUL predictions, their exclusive focus on temporal attention within multivariate time series sensor readings, without considering sensor-wise attention, raises concerns about potential inaccuracies in RUL predictions. To address this concern, our paper proposes a novel solution in the form of a two-stage attention-based hierarchical Transformer (STAR) framework. This approach incorporates a two-stage attention mechanism, systematically addressing both temporal and sensor-wise attentions. Furthermore, we enhance the STAR RUL prediction framework by integrating hierarchical encoder–decoder structures to capture valuable information across different time scales. By conducting extensive numerical experiments with the CMAPSS datasets, we demonstrate that our proposed STAR framework significantly outperforms the current state-of-the-art models for RUL prediction.

## 1. Introduction

As modern sensor technologies advance and automation continues to rise, the shift from traditional preventive maintenance (PM) to condition-based predictive maintenance (CBPM) marks a significant evolution in aviation management. Traditional PM relies on routine inspections and repairs following a predetermined schedule, often leading to unnecessary maintenance activities and associated costs. In contrast, CBPM leverages advanced technologies such as sensors and data analytics to monitor real-time equipment conditions. This proactive approach allows organizations to make informed decisions by predicting potential failures or maintenance needs, optimizing schedules, minimizing downtime, and reducing overall costs [1,2]. The transition to CBPM represents a more efficient and proactive strategy, enhancing the reliability and performance of critical assets while maximizing operational efficiency.

Central to the CBPM methodology is the prediction of the remaining useful life (RUL), an extremely challenging task that has attracted considerable interest from the research community in recent years. The RUL prediction poses significant challenges due to the complex nature of the underlying systems and the dynamic operational conditions they endure. For example, for RUL prediction, turbofan engines exhibit non-linear degradation patterns over time, and the relationship between sensor readings and the health state of the engine is often intricate. Also, the degradation of turbofan engines can be highly sensitive to operational profiles. Small variations in operating conditions can lead to different rates of degradation. Developing a model that is robust to these variations and accurately captures the sensitivity to different profiles is a challenging aspect of RUL prediction. The objective of RUL prediction is to accurately estimate the time span between the current moment and the projected conclusion of a system’s operational life cycle. This estimation serves as a crucial input for subsequent maintenance scheduling, enabling proactive and timely maintenance actions.

Conventional methods for estimating RUL encompass two main approaches: physics-based methods and statistics-based methods. Physics-based methods employ mathematical tools such as differential equations to model the degradation process of a system, offering insights into the physical mechanisms governing its deterioration [3,4,5,6,7,8,9,10]. On the other hand, statistics-based methods rely on probabilistic models, such as the Bayesian hidden Markov model (HMM), to approximate the underlying degradation process [11,12,13,14,15,16]. Nevertheless, these conventional methods either depend on prior knowledge of system degradation mechanics or rest on probabilistic assumptions about the underlying statistical degradation processes. The inherent complexity of real-world degradation processes poses a significant challenge in accurately modeling them. Consequently, the application of these methods in real-world CBPM systems may lead to suboptimal prediction performance and less effective decisions in maintenance scheduling.

To overcome the limitations of traditional physics-based and statistics-based methods, researchers are redirecting their focus towards the adoption of artificial intelligence and machine learning (AI/ML) techniques for predicting the RUL. This strategic shift has been prompted by the demonstrated successes of AI/ML applications in diverse domains, including but not limited to cybersecurity [17,18], engineering [19,20], and geology [21,22]. The growing prevalence of data and the continuous advancements in computational power further underscore the potential of AI/ML in increasing the accuracy of RUL prediction. This trend offers a promising avenue for overcoming the inherent limitations associated with traditional methodologies. Following this trend, our study introduces the STAR framework, an AI/ML-driven approach that combines a two-stage attention mechanism and integrates a hierarchical encoder–decoder structure for predicting the RUL.

The rest of the paper is structured as follows: Section 2 provides a review of related work in AI/ML-based RUL prediction. Section 3 provides a comprehensive exposition of the STAR model architecture. Section 4 intricately explores the experimental details, presents the results, and offers a thorough analysis. Finally, Section 5 concludes the paper.

## 2. Related Literature

Deep learning (DL) stands out as the most widely employed AI/ML approach, demonstrating remarkable success across diverse disciplines and delivering substantial performance enhancements when compared with traditional methods [23]. Recurrent neural networks (RNNs) and convolutional neural networks (CNNs) stand out as widely employed DL methodologies for RUL prediction, leveraging their abilities in capturing temporal patterns and spatial features in multidimensional time series data. Peng et al. [24] proposed a method that combines a one-dimensional CNN with fully convolutional layers (1-FCCNN) and a long short-term memory (LSTM) network to predict the RUL for turbofan engines. Remadna et al. [25] developed a hybrid approach for RUL estimation combining CNNs and bidirectional LSTM (BiLSTM) networks to extract spatial and temporal features sequentially. Hong et al. [26] and Nair et al. [27] developed an LSTM model, achieving heightened accuracy, while addressing challenges of dimensionality and interpretability using dimensionality reduction and Shapley additive explanation (SHAP) techniques [28]. Rosa et al. [29] introduced a generic fault prognosis framework employing LSTM-based autoencoder feature learning methods, emphasizing the semi-supervised extrapolation of reconstruction errors to address imbalanced data in an industrial context. Ji et al. [30] proposed a hybrid model for accurate airplane engine failure prediction, integrating principal component analysis (PCA) for feature extraction and BiLSTM for learning the relationship between the sensor data and RUL. Peng et al. [31] introduced a dual-channel LSTM neural network model for predicting the RUL of machinery, addressing challenges related to noise impact in complex operations and diverse abnormal environments. Their proposed method adaptively selects and processes time features, incorporates first-order time feature information extraction using LSTM, and creatively employs a momentum-smoothing module to enhance the accuracy of RUL predictions. Similarly, Zhao et al. [32] designed a double-channel hybrid prediction model for efficient RUL prediction in industrial engineering, combining a CNN and BiLSTM network to address drawbacks in spatial and temporal feature extraction. Wang et al. [33] addressed challenges in RUL prediction by introducing a novel fusion model, B-LSTM, combining a broad learning system (BLS) for feature extraction and LSTM for processing time series information. Yu et al. [34] presented a sensor-based data-driven scheme for system RUL estimation, incorporating a bidirectional RNN-based autoencoder and a similarity-based curve matching technique. Their approach involves converting high-dimensional multi-sensor readings into a one-dimensional health index (HI) through unsupervised training, allowing for effective early-stage RUL estimation by comparing the test HI curve with pre-built degradation patterns.

While RNNs and CNNs have demonstrated effectiveness in RUL estimation, they come with certain limitations. RNNs, due to their sequential nature, may suffer from slow training and prediction speeds, particularly when dealing with long sequences of time series data. The vanishing gradient problem in RNNs can impede their ability to capture dependencies across extended time intervals, potentially leading to the inadequate modeling of degradation patterns. Additionally, RNNs may struggle with incorporating contextual information from distant time steps, limiting their effectiveness in capturing complex temporal relationships. On the other hand, CNNs, designed for spatial feature extraction, may overlook temporal dependencies crucial in RUL prediction tasks, potentially leading to suboptimal performance.

The Transformer architecture [35], initially introduced for natural language processing tasks, represents a paradigm shift in sequence modeling. Unlike traditional models like RNNs and CNNs, Transformers rely on a self-attention mechanism, enabling the model to weigh the importance of different elements in a sequence dynamically. This attention mechanism allows Transformers to capture long-range dependencies efficiently, overcoming the vanishing gradient problem associated with RNNs. Moreover, Transformers support the parallelization of computation, making them inherently more scalable than sequential models like RNNs. The self-attention mechanism in Transformers also addresses the challenges faced by CNNs in capturing temporal dependencies in sequential data, as it does not rely on fixed receptive fields.

Within the realm of RUL prediction, numerous studies have introduced diverse customized Transformer architectures tailored specifically for RUL estimation. By utilizing a Transformer encoder as the central component, Mo et al. [36] presented an innovative method for predicting the RUL in industrial equipment and systems. The model proposed tackles constraints found in RNNs and CNNs, providing adaptability to capture both short- and long-term dependencies, facilitate parallel computation, and integrate local contexts through the inclusion of a gated convolutional unit. Introducing the dynamic length Transformer (DLformer), Ren et al. [37] proposed an adaptive sequence representation approach, acknowledging that individual time series may require different sequence lengths for accurate prediction. The DLformer achieves significant gains in inference speed, up to 90%, while maintaining a minimal degradation of less than 5% in model accuracy across multiple datasets. Zhang et al. [38] introduced an enhanced Transformer network tailored for multi-sensor signals to improve the decision-making process for preventive maintenance in industrial systems. Addressing the limitations of existing Transformer models, the proposed model incorporates the Trend Augmentation Module (TAM) and Time-Feature Attention Module (TFAM) into the traditional Transformer architecture, demonstrating superior performance in various numerical experiments.

Li et al. [39] introduced an innovative approach to enhance the RUL prediction accuracy using a novel encoder–decoder architecture with Gated Recurrent Units (GRUs) and a dual attention mechanism. Integrating domain knowledge into the attention mechanism, their proposed method simultaneously emphasizes critical sensor data through knowledge attention and extracts essential features across multiple time steps using time attention. Peng et al. [40] developed a multiscale temporal convolutional Transformer (MTCT) for RUL prediction. The unique features of MTCT include a convolutional self-attention mechanism incorporating dilated causal convolution for improved global and local modeling and a temporal convolutional network attention module for enhanced local representation learning. Xiang et al. [41] introduced the Bayesian Gated-Transformer (BGT) model, a novel approach for RUL prediction with a focus on reliability and quantified uncertainty. Rooted in the Transformer architecture and incorporating a gated mechanism, the BGT model effectively quantifies both epistemic and aleatory uncertainties and providing risk-aware RUL predictions. Most recently, Fan et al. [42] introduced the BiLSTM-DAE-Transformer framework for RUL prediction, utilizing the Transformer’s encoder as the framework’s backbone and integrating it with a self-supervised denoising autoencoder that employs BiLSTM for enhanced feature extraction.

Although Transformer-based methods for RUL prediction outperform traditional RNNs and CNNs, they are not without their limitations. Firstly, in the application of the self-attention mechanism to time series sensor readings for RUL prediction, these methods emphasize the weights of distinct time steps while overlooking the significance of individual sensors within the data stream—an aspect critical for comprehensive prediction performance. Secondly, in the utilization of temporal self-attention, these methods treat sensor readings within a single time step as tokens. However, a single time step reading usually has few semantic meanings. Consequently, a singular focus on the attention of individual time steps proves inadequate for capturing nuanced local semantic information requisite for RUL prediction. Inspired by recent advances in multivariate time series prediction, particularly those aimed at improving accuracy through the incorporation of both temporal and variable attention [43,44,45], we introduce the STAR framework to tackle these challenges. The proposed framework integrates a two-stage attention mechanism, sequentially capturing temporal and sensor-specific attentions, and incorporates a hierarchical encoder–decoder structure designed to encapsulate temporal information across various time scales. As demonstrated later in our numerical experiments, the proposed STAR framework consistently outperforms existing RUL prediction models. This superior performance has significant implications for maintenance scheduling, enabling more effective and timely proactive maintenance actions within the context of CBPM.

The studies conducted in [46,47] share some similarities with our current research. Notably, they also integrate sensor-wise attention into the prediction process. However, these approaches treat temporal attention and sensor-wise variable attention as independent entities. In other words, they generate two copies of the input sensor readings: one for computing temporal attention and the other for calculating sensor-wise variable attention. Subsequently, a fusion layer is employed to combine these two forms of attention together. In contrast to their methodology, our approach takes a distinct route by utilizing a two-stage attention mechanism. Our approach sequentially captures temporal attention and sensor-wise variable attention, addressing each aspect separately. This two-stage attention strategy is designed to provide a nuanced understanding of both temporal dynamics and individual sensor contributions for more comprehensive prediction capabilities.

The main contributions of this work are as follows:

1. We introduce a two-stage attention mechanism that sequentially captures both temporal attention and sensor-wise variable attention, distinguishing our approach from existing methods that handle these attentions separately and independently. This marks the first successful application of such a mechanism to turbofan engine RUL prediction. Our experimental results substantiate the effectiveness of our approach, showcasing superior prediction accuracy compared to existing methods.
2. We propose a hierarchical encoder–decoder framework to capture temporal information across various time scales. While multiscale prediction has shown superior performance in numerous computer vision and time series classification tasks [45,48], our work marks the first successful implementation of multiscale prediction in RUL prediction. Additionally, our encoder–decoder architecture diverges from existing Transformer-based RUL prediction models, which typically incorporate only a Transformer encoder in their prediction modeling. Our numerical experiments reveal that the inclusion of the encoder–decoder architecture leads to notable improvements in prediction performance.

## 3. Methodology

**Figure 1:**
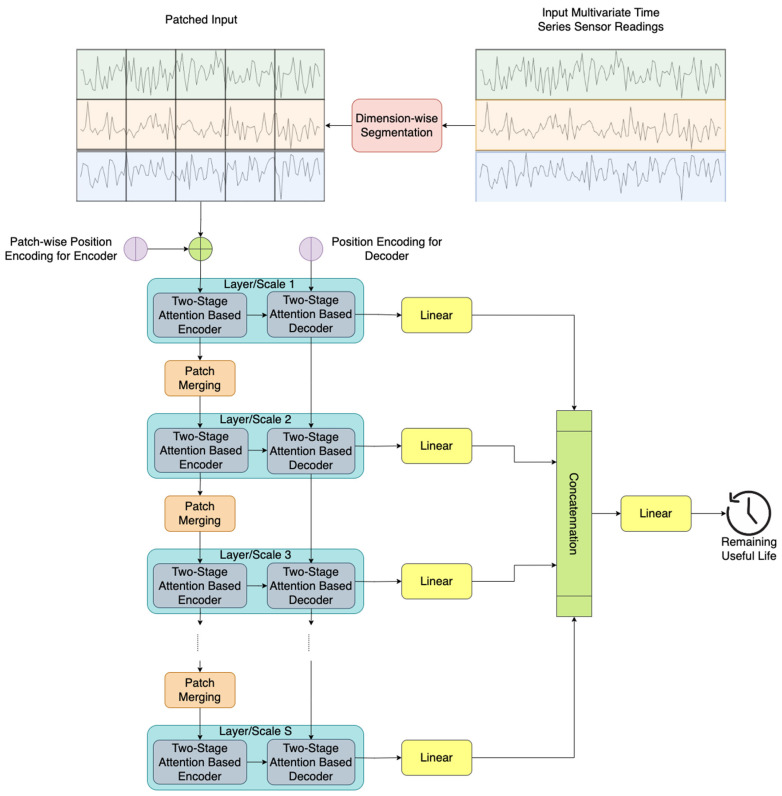
Overall structure of the proposed STAR frameworks.

Our study is dedicated to predicting the RUL of a turbofan engine based on historical multivariate time series sensor readings denoted as $x_{1:T}\in R^{T\times D}$, where T represents the number of time steps in the input data, and D is the number of onboard sensors. The proposed STAR framework, illustrated in Figure 1, comprises five key components:

1. Dimension-wise segmentation and embedding (Section 3.1): Each sensor’s univariate time series is segmented into K disjoint patches with length L. To embed individual patches, a combination of an affine transformation and positional embedding is utilized [35].
2. Encoder (Section 3.2): adapting the traditional Transformer encoder [35], we introduce a modification that integrates a two-stage attention mechanism to capture both temporal and sensor-wise attentions.
3. Decoder (Section 3.3): refining the conventional Transformer decoder [35], our modification introduces a two-stage attention mechanism aimed at capturing both temporal and sensor-wise attentions.
4. Patch merging (Section 3.4): merging neighboring patches for each sensor in the temporal domain facilitates the creation of a coarser patch segmentation, enabling the capture of multiscale temporal information.
5. Prediction layer (Section 3.5): the final RUL prediction is achieved by concatenating information across different time scales through the use of a multi-layer perceptron (MLP).

As depicted in Figure 1, our proposed framework takes multivariate time series sensory readings as the input. Subsequently, the model processes the patched input obtained through the dimension-wise segmentation of these multivariate time series for prediction. This patched input undergoes positional embedding and enters a two-stage attention-based encoder for feature encoding. It is important to note that the encoder’s output represents the finest feature representation of the patched input in a time scale. Subsequently, the output proceeds to a two-stage attention-based decoder block for prediction at the finest scale, and a patch merging block facilitates the generation of a coarser representation in time scale.

The subsequent subsections elaborate on each of the above five components.

### 3.1. Dimension-Wise Segmentation and Embedding

The original development of the Transformer architecture focused on natural language processing tasks like neural machine translation [35,49]. Consequently, when applied to time series prediction tasks, the conventional approach treats each time step in the time series data as a token, akin to the treatment of words in natural language processing tasks. However, the information contained in a single time step is often limited, potentially resulting in suboptimal performance for time series prediction tasks. Inspired by the recent success of using Transformers in computer vision tasks, where input image data are segmented into small patches, researchers in time series predictions have adopted a similar segmentation procedure, leading to enhanced performance in time series prediction tasks [43,44,45]. In line with this approach, we employ a similar segmentation procedure in our work for RUL prediction.

The dimension-wise segmentation segments each sensor time series reading into K smaller disjoint patches with length L, as shown in the top left of Figure 1. Each segmentation is denoted as $x_{k,d}\in R^{L}$ (k=1,…,K, d=1,…, D) and embedded with an affine transformation and positional encoding:

(1) $$x_{k,d}^{\left(e\right)}=A\cdot x_{k,d}+E_{k,d}$$

where $A\in R^{d_{model}\times L}$ is a learnable matrix for embedding and $E_{k,d}\in R^{d_{model}}$ denotes the learnable positional encoding for each patch. As a result, the information of the original patch $x_{k,d}$ is embedded into a $d_{model}$ dimensional space.

### 3.2. Two-Stage Attention-Based Encoder

**Figure 2:**
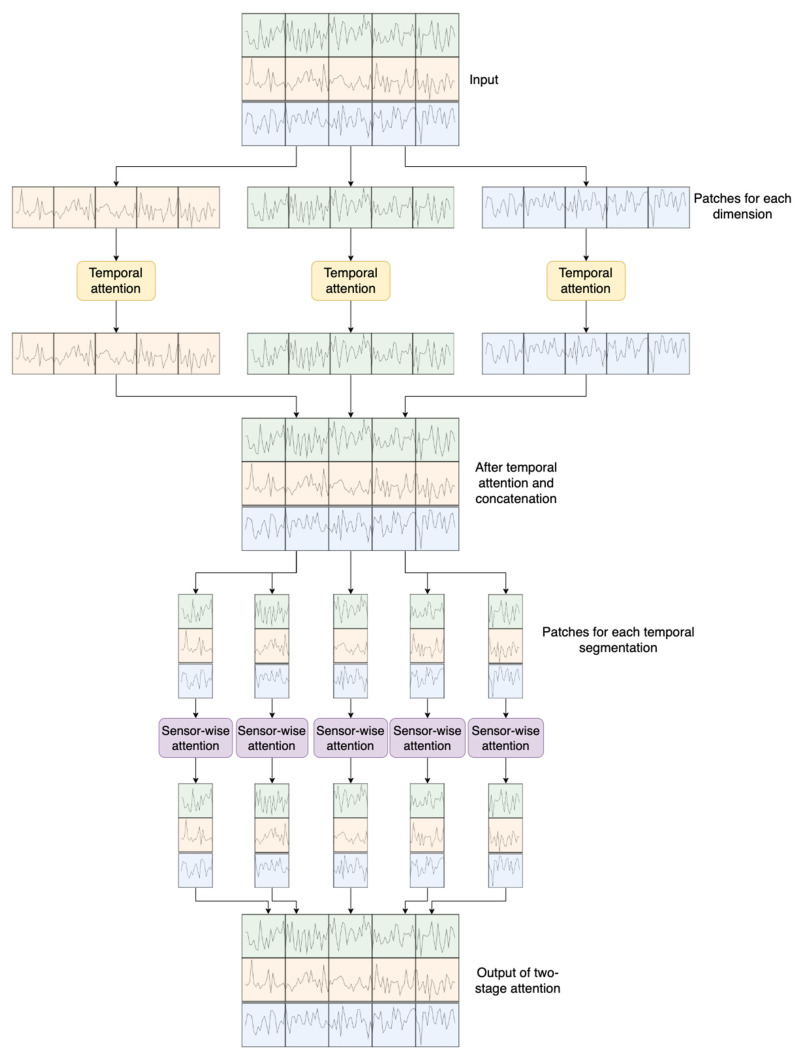
Two-stage attention-based encoder.

Denote $X^{\left(e\right)}\in R^{K\times D\times d_{model}}$ as the embedded inputs, which act as the input for the encoder, as depicted at the top of Figure 2.

**Figure 3:**
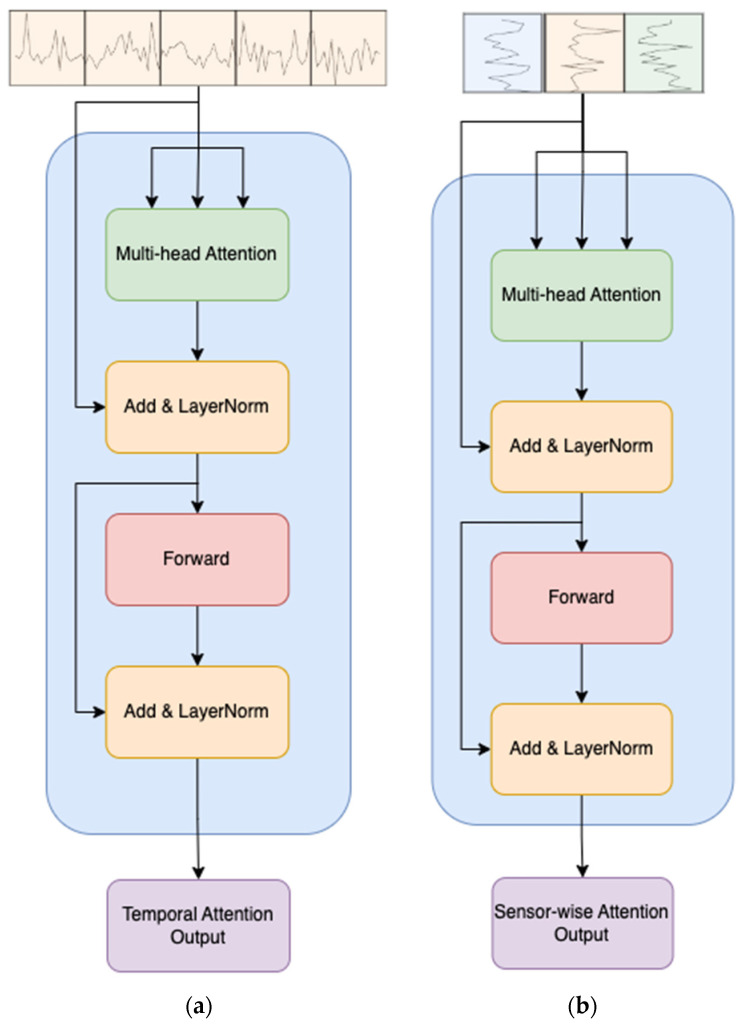
Temporal and sensor-wise variable attentions. (**a**) Network architecture for temporal attention. (**b**) Network architecture for sensor-wise variable attention.

The input is initially partitioned into D distinct fragments. Each fragment $X_{:,\;d,:}^{\left(e\right)}$ is then fed into the temporal attention calculation block, closely resembling the conventional multi-head self-attention (MSA) [35], as depicted in Figure 3a. This block is responsible for capturing temporal dependencies within each sensor’s readings.

MSA is a critical mechanism in the Transformer architecture, particularly beneficial for tasks involving sequential data processing. In the original Transformer formulation, the self-attention mechanism is enhanced by introducing multiple attention heads. This extension allows the model to attend to different positions in the input sequence simultaneously and learn diverse relationships between elements.

The standard self-attention mechanism computes attention scores using the following equation for a single attention head:

(2) $$Attention\left(Q,K,V\right)=softmax\left(\frac{QK^{T}}{\sqrt{d_{k}}}\right)V$$

Here, Q, K, and V denote the query, key, and value matrices, respectively. The softmax operation normalizes the attention scores, and $d_{k}$ is a scaling factor to control the magnitude of the scores. The resulting attention values are then multiplied by the value matrix to obtain the weighted sum.

In the multi-head attention mechanism, the process is parallelized across h attention heads, each with distinct learned linear projections of the input Q, K, and V matrices. The final output is obtained by concatenating the outputs from all attention heads with a linear transformation:

(3) $$MSA\left(Q,K,V\right)=Concat\left(head^{1},head^{2},\ldots ,head^{h}\right)W_{o}$$

Here, $W_{o}$ is a learned linear transformation matrix applied to the concatenated outputs. Then, the temporal attention block can be expressed as follows:

(4) $${\hat{X}}_{:,d,:}^{\left(e\right)}=LayerNorm\left(X_{:,d,:}^{\left(e\right)}+MSA\left(X_{:,d,:}^{\left(e\right)},X_{:,d,:}^{\left(e\right)},X_{:,d,:}^{\left(e\right)}\right)\right)$$

(5) $$X_{:,d,:}^{temp}=LayerNorm\left({\hat{X}}_{:,d,:}^{\left(e\right)}+Forward\left({\hat{X}}_{:,d,:}^{\left(e\right)}\right)\right)$$

where LayerNorm denotes layer normalization and Forward represents a feedforward network. These procedures are widely utilized in network architectures based on Transformers [35,44,46]. Following the temporal attention block, $X^{temp}\in R^{K\times D\times d_{model}}$ is subsequently fed into the sensor-wise attention block, depicted in Figure 3b, to capture sensor-wise attention. The computation within the sensor-wise attention block is analogous to that of the temporal attention block, utilizing the input $X_{k,:,:}^{temp}$. This mechanism allows the model to attend to important sensors and capture relevant features in the context of the temporal sequence.

### 3.3. Patch Merging

**Figure 4:**
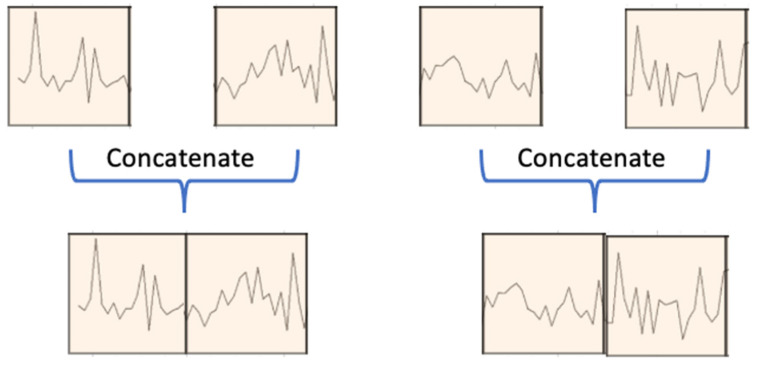
Patch merging.

As illustrated in Figure 1, the output of the two-stage attention-based encoder, denoted as $X^{enc,s}$, undergoes processing in the patch merging block to generate coarser patches, facilitating multiscale predictions. Specifically, in the patch merging block (see Figure 4), adjacent patches for each sensor are combined in the time domain, creating a coarser patch segmentation. These resultant coarser patches serve as the input for the subsequent layer/scale (s+1) in the encoder. This hierarchical structure enables the model to capture temporal information across different time scales, enhancing its predictive capabilities.

The concatenated coarser patch undergoes an affine transformation to maintain the dimensionality at $d_{model}$. The procedure is summarized by the equation below:

(6) $$X_{i}^{enc,s+1}=B\cdot \left[X_{2i,d}^{enc,s},\;X_{2i+1,d}^{enc,s}\right]$$

Here, $B\in R^{d_{model}\times 2d_{model}}$ represents a learnable matrix employed for dimensionality preservation during the patch merging process.

### 3.4. Two-Stage Attention-Based Decoder

**Figure 5:**
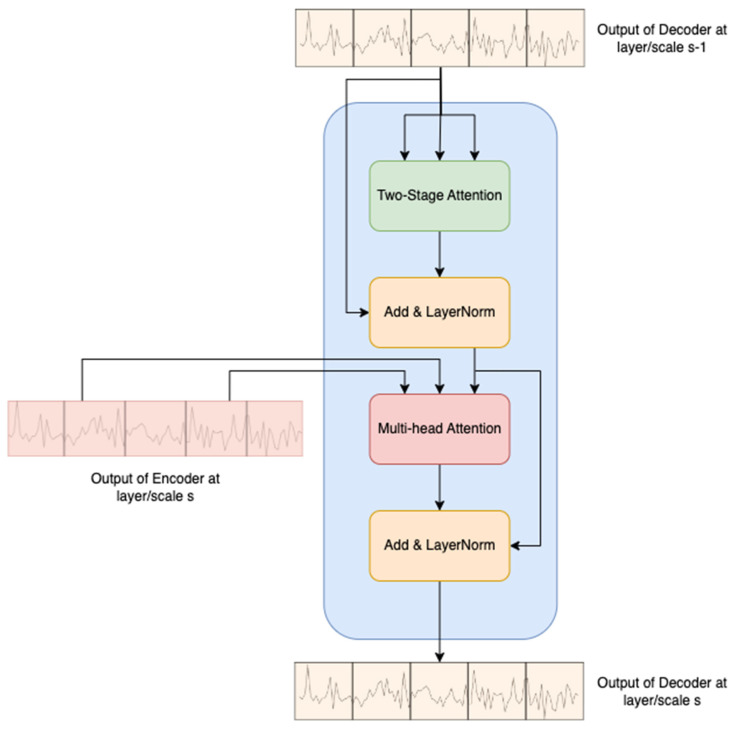
Two-stage attention-based decoder.

At layer/scale s, the inputs of the two-stage attention-based decoder are $X^{enc,s}$ and $X^{dec,s-1}$, where $X^{dec,s-1}$ is the output of the decoder from the previous layer/scale s−1. The decoder architecture closely resembles that of the original Transformer network, with the modification of replacing the masked multi-head self-attention (MMSA) with a two-stage attention mechanism, as illustrated in Figure 5.

In the decoder process, the output of the decoder at the previous layer s−1 undergoes the two-stage attention block, followed by a residual connection and layer normalization. Subsequently, the output of the encoder in the current layer s serves as the keys and values for the MSA block. This modification enhances the decoder’s ability to capture both temporal and sensor-wise attention, contributing to improved RUL prediction accuracy. It is important to note that the input of the decoder at the initial layer/scale comprises a fixed positional encoder defined by trigonometric functions, as introduced by Vaswani et al. [35].

### 3.5. Prediction Layer

As depicted in the right-hand part of Figure 1, the outputs of the decoders at different layers/scales are fed into separate MLPs to further embed the information, enhancing the model’s ability to capture intricate patterns for RUL prediction. The outputs from these individual MLP blocks are then concatenated and passed into another MLP to make the final prediction. This hierarchical embedding and fusion process enables the model to capture both local and global dependencies, contributing to improved accuracy in predicting the RUL of turbofan engines.

## 4. Experimental Results and Analysis

The experiments were performed on a computational system comprising an Intel Core i9 3.6 GHz processor, 64 GB of RAM and 4 NVIDIA RTX 3080 GPU.

In the following subsections, we will initially present the CMAPSS dataset utilized in our experiments and discuss data preprocessing in Section 4.1. Subsequently, Section 4.2 will delve into the details of hyperparameter tuning and implementation specifics. The performance metrics employed to evaluate the proposed STAR framework are introduced in Section 4.3. The performance results of the STAR framework will be presented and compared with several existing benchmarks in Section 4.4. Finally, in Section 4.5, a set of ablation studies is conducted to demonstrate the importance of each component in our STAR framework.

### 4.1. Data and Preprocessing

**Figure 6:**
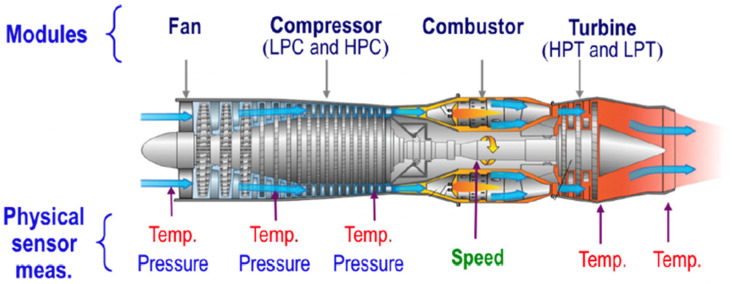
Turbofan engine model [50].

We opted to utilize the NASA Commercial Modular Aero-Propulsion System Simulation (CMAPSS) dataset as the benchmark for assessing our model. Developed by NASA, CMAPSS is an extensive simulation framework designed to replicate the behavior of commercial aircraft turbofan engines, facilitating detailed investigations into engine performance, diagnostics, and prognostics. Widely recognized in the field of Prognostics and Health Management (PHM) for aircraft turbofan engines, the CMAPSS dataset is generated within this simulation environment, providing a valuable repository of multivariate time series data. Simulating the operation of a fleet of engines under diverse conditions and fault scenarios, the dataset includes sensor readings from various engine components. Researchers leverage this resource to explore and devise methods for tasks such as RUL prediction, fault diagnosis, and performance analysis. Figure 6 illustrates the structure of a turbofan engine within CMAPSS, comprising five modules: fan, low-pressure turbine (LPT), high-pressure turbine (HPT), low-pressure compressor (LPC), and high-pressure compressor (HPC).

Table 1 below describes the composition of the C-MAPSS data, comprising four distinct sub-datasets. These datasets cover various operational conditions and fault modes, each containing both training and testing engines, serving the purpose of model training and evaluation.

Each observation in the dataset is a snapshot of data taken during a single operating time cycle with 21 onboard sensors monitoring the engine’s health status, as detailed in Table 2.

However, not all sensors contribute useful information for RUL prediction, as some remain constant until failure [36,40,47]. Following the approach outlined in [36], we selectively incorporate data from 14 sensors (sensors 2, 3, 4, 7, 8, 9, 11, 12, 13, 14, 15, 17, 20, 21) into our training and testing processes. Additionally, we apply max–min normalization to the sensor readings, which is expressed by the formula [36]:

(7) $$x^{\prime }=\frac{x-x_{min\;}}{x_{max}-x_{min}}$$

Here, x represents the original sensor readings, $x_{min\;}$ is the minimum value of the sensor readings, and $x_{max\;}$ is the maximum value of the sensor readings. This normalization technique scales the sensor values to a consistent range [0, 1], promoting uniformity and aiding in the training process for effective RUL prediction models. The selective inclusion of sensors and normalization contribute to improved model performance and robustness [51].

In traditional RUL estimation, the common practice involves assigning target values that decrease linearly with time, assuming a linear degradation of the system’s health over its operational life. However, this simplified assumption may not accurately reflect the real-world behavior of system degradation, especially during the initial stages when degradation is typically negligible. To address this limitation, our approach, inspired by a piece-wise linear RUL target function proposed in previous studies [52,53], introduces a more nuanced labeling strategy for the RUL in the CMAPSS datasets. In our approach, the RULs are initially labeled with a constant value ($RUL_{max}$), representing a phase of minimal degradation. Subsequently, the system enters a phase of linear degradation until it reaches failure. This truncated linear model better captures the complex evolution of the RUL, considering varying degradation rates over different life cycle phases. By aligning our RUL labeling with the actual behavior of turbofan engines, our method provides a more realistic representation of system health progression, especially during the initial stages of operation.

### 4.2. Hyperparameter Tuning

The hyperparameter tuning process for the proposed STAR model involves an extensive grid search to identify the optimal configuration in terms of the root mean squared error (RMSE) [54]. The grid search encompasses key hyperparameters, such as the learning rate, batch size, optimizer, input time series length, the number of layers/scales for multiscale prediction, and the dimension of embedding space and number of heads in MSA [46,55]. A detailed breakdown of the possible range and grid for these hyperparameters is provided in Table 3. This grid search methodology allows for a comprehensive examination of various parameter combinations, facilitating the identification of the most effective setup for RUL prediction.

The optimal hyperparameter combinations for FD001 to FD004 are presented in Table 4. Subsequently, the prediction model is instantiated using these sets of hyperparameters to predict the RUL for testing engines.

It is evident from the results that the FD002 and FD004 datasets necessitate a longer time series length, a greater number of layers/scales, and more heads in the MSA compared to the FD001 and FD003 datasets. We posit that this disparity arises from the fact that the FD002 and FD004 datasets are simulated under diverse operational conditions. Consequently, they demand a more intricate network structure to extract valuable features for RUL prediction. Additionally, these datasets require longer input sequences containing more information to generate accurate predictions.

### 4.3. Evaluation Metric

In evaluating the predictive performance of the proposed model for the RUL, two key metrics are employed: the RMSE and an effectiveness Score. The RMSE, expressed by Equation (8) [56], is a widely used metric in RUL estimation evaluation, providing equal penalty weights for both the underestimation and overestimation of the RUL. It calculates the square root of the mean squared differences between the true RUL values $y_{i}$ and the predicted RUL values ${\hat{y}}_{i}$.

(8) $$RMSE=\sqrt{\frac{\sum_{i=1}^{n}{\left(y_{i}-{\hat{y}}_{i}\right)}^{2}}{N}}$$

On the other hand, the effectiveness Score, defined by Equation (9) [50], introduces distinct penalty weights for the direction of prediction deviation. The Score penalizes advancements (where ${\hat{y}}_{i}$ is smaller than $y_{i}$) with a smaller coefficient, recognizing the opportunity for proactive maintenance planning. Conversely, when predictions lag (where ${\hat{y}}_{i}$ is larger than $y_{i}$), a larger penalty coefficient is applied, reflecting the potential for more severe consequences when the maintenance is performed too late.

(9) $$Score=\left\{\begin{array}{c} \overset{N}{\underset{i=1}{\sum }}e^{-\left(\frac{y_{i}-{\hat{y}}_{i}}{13}\right)}-1,\;\;\;d<0\\ \overset{N}{\underset{i=1}{\sum }}e^{-\left(\frac{y_{i}-{\hat{y}}_{i}}{10}\right)}-1,\;\;\;d\geq 0 \end{array}\right.$$

### 4.4. RUL Prediction

In this section, we rigorously evaluate the performance of the proposed STAR framework for RUL prediction. To benchmark its effectiveness, we compare the proposed model against a suite of existing methods widely recognized in the field. These methods include BiLSTM [57], the gated convolutional Transformer (GCT) [36], DCNN [58], ELSTMNN [59], DATCN [60], AGCNN [61], BiLSTM attention model [62], DAST [46], DLformer [37], 1D-CNN-LSTM [63], CNN-LSTM-SAM [64], and BiLSTM-DAE-Transformer [42]. Table 5 shows the comparison results.

As presented in Table 5, the proposed STAR framework consistently outperforms existing RUL prediction models across all the datasets, showcasing its superior predictive capabilities. Notably, for the FD001 and FD002 datasets, our method demonstrates the best performance, achieving the lowest RMSE and Score values. Remarkably, the STAR framework exhibits significant improvements in both the RMSE and Score metrics for the challenging FD002 dataset, surpassing the state-of-the-art models by 12% and 15% in terms of the RMSE and Score, respectively. This highlights the effectiveness of capturing sensor-wise attention, which is particularly crucial in cases such as FD002, simulated under diverse operating conditions. For the FD003 dataset, our STAR framework attains the best performance in terms of the RMSE and the second-best performance in terms of the Score. This observation suggests a tendency to underestimate the RUL for this dataset, leading to a larger penalty when calculating the Score metric. Consequently, while our model excels when evaluated based on the RMSE, there is a slight deviation when employing the Score metric. Contrarily, for the FD004 dataset, the trends are reversed compared to FD003. In this scenario, our model achieves the second-best performance in terms of the RMSE while securing the top position in the Score. It is noteworthy that the difference in the RMSE between our method and the best model for FD004 (DLformer) is only 0.01, highlighting the competitive performance of the STAR framework. Another interesting observation in Table 5 is the consistent outperformance of the Transformer-based models (DAST, DLformer, BiLSTM-DAE-Transformer, and STAR) compared to other existing CNN- and RNN-based models. Notably, our two-stage attention-based STAR framework exhibits the best performance among these single-stage Transformer-based models, achieving better results in terms of both the RMSE and Score across nearly all the CMAPSS datasets.

**Figure 7:**
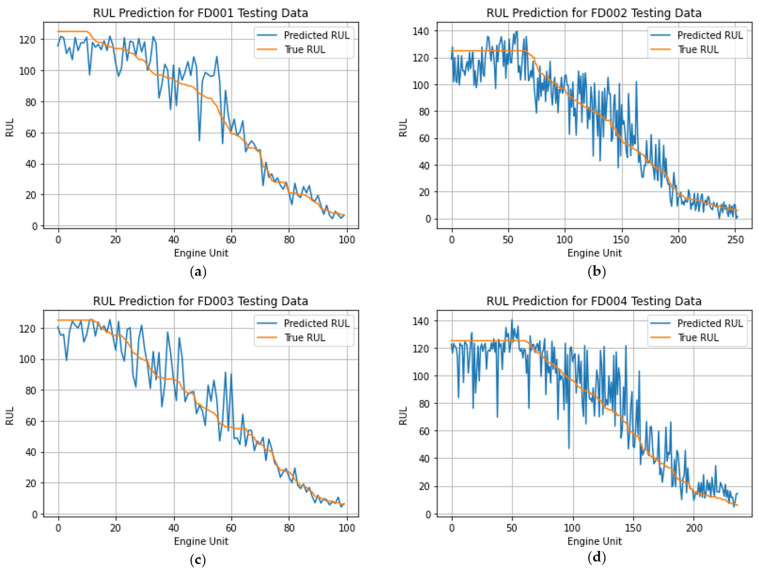
Results for comparing estimated RUL and actual RUL. (**a**) Comparison result for FD001; (**b**) comparison result for FD002; (**c**) comparison result for FD003; (**d**) comparison result for FD004.

Figure 7 serves as a comprehensive visual representation, offering a detailed comparison between the predicted RUL generated by our STAR model and the ground truth RULs across the FD001 to FD004 testing datasets. The *x*-axis corresponds to the Engine Unit Index, while the *y*-axis depicts the RUL. The graphical depiction provides insights into the model’s performance under varying conditions. 

For enhanced clarity in visualization, we adhere to the conventional practice of arranging all the test sequences along the *x*-axis in ascending order based on their ground truth RUL. In Figure 7a,c, the model exhibits notable precision, especially for scenarios where the ground truth RUL is relatively small (below 60). However, for the FD002 and FD004 datasets, the prediction results display a discernible level of noise compared to the smoother outcomes observed in FD001 and FD003. This observed variability may be attributed to differences in operational complexities, as evidenced by the varying numbers of operating conditions and fault modes, along with the size of the training dataset. Notably, FD002 and FD004 involve simulations under six distinct operating conditions, while FD001 and FD003 are conducted under a single operating condition. The heightened complexity in FD002 and FD004 likely contributes to the observed noise in predictions, underscoring the model’s sensitivity to the intricacies of working conditions and the dataset size across diverse scenarios.

### 4.5. Ablation Study

In this section, we conduct ablation experiments to assess the impact of individual components in our proposed model. Specifically, we compare the prediction performances, evaluated in terms of the RMSE, for the following models, all utilizing the same set of hyperparameters selected from Table 4:

- STAR: the proposed model with a two-stage attention mechanism and hierarchical encoder–decoder.
- STAR-Temporal: the proposed model with temporal attention only and a hierarchical encoder–decoder.
- STAR-SingleScale: the proposed model with a two-stage attention mechanism and hierarchical encoder–decoder, excluding the patch merging step between different layers/scales, as depicted in Figure 1.

The findings revealed in Table 6 emphasize the importance of each component in our proposed STAR model, shedding light on their respective contributions to achieving a noteworthy prediction performance. Notably, the STAR model without sensor-wise variable attention and multiscale information exhibits a decline in the prediction performance, particularly evident in the case of the more complex FD002 and FD004 datasets.

## 5. Conclusions

This paper presents an innovative STAR framework designed for predicting the RUL of turbofan engines. Leveraging a two-stage attention mechanism, our proposed model adeptly captures both temporal and sensor-wise variable attention. By utilizing a hierarchical encoder–decoder structure to integrate multiscale information, the model produces hierarchical predictions, demonstrating superior performance in predicting the RUL. Using the CMAPSS dataset, we illustrate the importance of incorporating both temporal attention and sensor-wise variable attention for RUL prediction through a series of numerical experiments. Notably, the STAR framework demonstrates clear improvements in both the RMSE and Score metrics for the challenging FD002 dataset, outperforming the state-of-the-art models by 12% and 15% in terms of the RMSE and Score, respectively. Additionally, for the FD001 dataset, our STAR framework outperforms existing algorithms by 4% and 10% in terms of the two metrics. The results highlight the promising potential of the STAR framework in achieving accurate and reliable RUL predictions, thereby contributing to advancements in prognostics for the health management of aircraft engines.

Despite the superior performance demonstrated by the proposed methods in predicting the RUL, it is important to note that the model inherently lacks the ability to provide explanations for its identification of equipment approaching failure. Therefore, a promising area for future research involves incorporating Explainable Artificial Intelligence (XAI) methods, such as SHAP and LIME, to unravel the prediction logic of the model. This enhancement has the potential to increase the applicability of the prediction model in practical scenarios, particularly within the context of CBPM. Additionally, it is noteworthy that the current STAR framework employs an attention mechanism with quadratic model complexity with respect to the length of input sequences. While this design proves effective for typical input lengths, it might become impractical when dealing with long input time series, which could contain more information than shorter sequences. Hence, an intriguing avenue for future research involves the development of methodologies specifically tailored for RUL prediction using longer multivariate time series as inputs.

## Figures and Tables

**Table 1:** Parameters of the C-MAPSS dataset.

Dataset	FD001	FD002	FD003	FD004
No. of Training Engines	100	260	100	249
No. of Testing Engines	100	259	100	248
No. of Operating Conditions	1	6	1	6
No. of Fault Modes	1	1	2	2

**Table 2:** C-MAPSS monitoring sensor data description [50].

Symbol	Description	Units
T2	Total temperature at fan inlet	R
T24	Total temperature at LPC inlet	R
T30	Total temperature at HPC inlet	R
T50	Total temperature at LPT inlet	R
P2	Pressure at fan inlet	psia
P15	Total pressure in bypass duct	psia
P30	Total pressure at HPC outlet	psia
Nf	Physical fan speed	rpm
Ne	Physical core speed	rpm
epr	Engine pressure ratio	-
Ps30	Static pressure at HPC outlet	psia
Phi	Ratio of fuel flow to Ps30	pps/psi
NRf	Corrected fan speed	rpm
NRe	Corrected core speed	rpm
BPR	Bypass ratio	-
farB	Burner fuel–air ratio	-
htBleed	Bleed enthalpy	-
Bf-dmd	Demanded fan speed	rpm
PCNfR-dmd	Demanded corrected fan speed	rpm
W31	HPT coolant bleed	lbm/s
W32	LPT coolant bleed	lbm/s

**Table 3:** Hyperparameters and ranges.

Hyperparameter	Range
learning rate	[0.0001, 0.01]
batch size	16, 32, 64
optimizer	Adam, SGD, RMSProp
time series length	32, 48, 64
number of layers/scales	1, 2, 3, 4
dimension of embedding space	128, 256, 512, 1024
number of heads for MSA	1, 2, 4, 6

**Table 4:** Best hyperparameter combinations for FD001, FD002, FD003, and FD004 datasets.

Hyperparameter	FD001	FD002	FD003	FD004
learning rate	0.0002	0.0002	0.0002	0.0002
batch size	32	64	32	64
optimizer	Adam	Adam	Adam	Adam
time series length	32	64	48	64
number of layers/scales	3	4	1	4
dimension of embedding space	128	64	128	256
number of heads for MSA	1	4	1	4

**Table 5:** Performance comparison. The bold number represents the best model, while the underscored number represents the second-best model. Methods include BiLSTM [56], GCT [36], DCNN [57], ELSTMNN [58], DATCN [59], AGCNN [60], BiLSTM attention model [61], DAST [46], DLformer [37], 1D-CNN-LSTM [62], CNN-LSTM-SAM [63], and BiLSTM-DAE-Transformer [42].

Method	FD001		FD002		FD003		FD004	
	RMSE	Score	RMSE	Score	RMSE	Score	RMSE	Score
BiLSTM (2018)	13.65	295	23.18	4130	13.74	317	24.86	5430
DCNN (2018)	12.61	237	22.36	1041	12.64	284	23.31	12,466
GCT (2021)	11.27	-	22.81	-	11.42	-	24.86	-
BiLSTM Attention (2021)	13.78	255	15.94	1280	14.36	438	16.96	1650
ELSTMNN (2021)	18.22	571	-	-	23.21	839	-	-
DATCN (2021)	11.78	229	16.95	1842	11.56	257	18.23	2317
AGCNN (2021)	12.42	225	19.43	1492	13.39	227	21.50	3392
DAST (2022)	11.43	203	15.25	924	11.32	**154**	18.23	1490
DLformer (2023)	-	-	15.93	1283	-	-	**15.86**	1601
1D-CNN-LSTM (2023)	16.1	437.2	-	-	-	-	**-**	-
CNN-LSTM-SAM (2023)	12.6	261	18.9	1156	12.5	253	20.5	2425
BiLSTM-DAE-Transformer (2023)	10.98	186	16.12	2937	11.14	252	18.15	3840
proposed method	**10.61**	**169**	**13.47**	**784**	**10.71**	202	15.87	**1449**

**Table 6:** Ablation study of the proposed STAR architecture.

Model	FD001	FD002	FD003	FD004
STAR	10.61	13.47	10.71	15.87
STAR-Temporal	11.62	16.67	12.01	18.44
STAR-SingleScale	12.33	16.11	12.49	17.71

## Data Availability

The data of this paper came from the NASA Prognostics Center of Excellence, and the data acquisition website was https://ti.arc.nasa.gov/tech/dash/groups/pcoe/prognostic-data-repository/#turbofan, accessed on 15 April 2023.
